# Automatic Segmentation of Bone Marrow Lesions on MRI Using a Deep Learning Method

**DOI:** 10.3390/bioengineering11040374

**Published:** 2024-04-12

**Authors:** Raj Ponnusamy, Ming Zhang, Yue Wang, Xinyue Sun, Mohammad Chowdhury, Jeffrey B. Driban, Timothy McAlindon, Juan Shan

**Affiliations:** 1 Department of Computer Science, Seidenberg School of CSIS, Pace University, New York City, NY 10038, USA; rp87701n@pace.edu (R.P.); yw46356p@pace.edu (Y.W.); mc32367p@pace.edu (M.C.); 2 Department of Computer Science, Boston University, Boston, MA 02215, USA; 3 School of Electronic Information and Electrical Engineering, Shanghai Jiao Tong University, Shanghai 200240, China; sunxinyuejn@126.com; 4 Department of Population and Quantitative Health Sciences, UMass Chan Medical School, Worcester, MA 01655, USA; jeffrey.driban@umassmed.edu; 5 Division of Rheumatology, Allergy, and Immunology, Tufts Medical Center, Boston, MA 02111, USA; tmcalindon@tuftsmedicalcenter.org

**Keywords:** knee osteoarthritis, bone marrow lesions, deep learning, segmentation, computer-aided diagnosis

## Abstract

Bone marrow lesion (BML) volume is a potential biomarker of knee osteoarthritis (KOA) as it is associated with cartilage degeneration and pain. However, segmenting and quantifying the BML volume is challenging due to the small size, low contrast, and various positions where the BML may occur. It is also time-consuming to delineate BMLs manually. In this paper, we proposed a fully automatic segmentation method for BMLs without requiring human intervention. The model takes intermediate weighted fat-suppressed (IWFS) magnetic resonance (MR) images as input, and the output BML masks are evaluated using both regular 2D Dice similarity coefficient (DSC) of the slice-level area metric and 3D DSC of the subject-level volume metric. On a dataset with 300 subjects, each subject has a sequence of 36 IWFS MR images approximately. We randomly separated the dataset into training, validation, and testing sets with a 70%/15%/15% split at the subject level. Since not every subject or image has a BML, we excluded the images without a BML in each subset. The ground truth of the BML was labeled by trained medical staff using a semi-automatic tool. Compared with the ground truth, the proposed segmentation method achieved a Pearson’s correlation coefficient of 0.98 between the manually measured volumes and automatically segmented volumes, a 2D DSC of 0.68, and a 3D DSC of 0.60 on the testing set. Although the DSC result is not high, the high correlation of 0.98 indicates that the automatically measured BML volume is strongly correlated with the manually measured BML volume, which shows the potential to use the proposed method as an automatic measurement tool for the BML biomarker to facilitate the assessment of knee OA progression.

## 1. Introduction

According to the Centers for Disease Control and Prevention’s arthritis factsheets, 58.5 million people in the United States have arthritis. It is a leading cause of work disability, resulting in annual costs for medical care and lost earnings of $303.5 billion [1,2]. By 2040, an estimated 78.4 million adults aged 18 years and older (25.9% of the projected total adult population) will have doctor-diagnosed arthritis, compared with 58.5 million adults in 2013–2015 [3]. Osteoarthritis (OA) is the dominant form of arthritis, and can cause severe pain and have an economic and social impact on both the patients and society. In general, OA is more likely to happen on weight-bearing joints, i.e., the most common site of OA is the knee. Bone marrow lesions (BMLs) are a form of subchondral inflammation seen as areas of increased signal intensity within bones that have been related to pain and progression of knee OA. BML volume is becoming a potential biomarker for knee OA evaluation and reducing its size could be part of the therapeutic goal [4,5].

**Figure 1:**
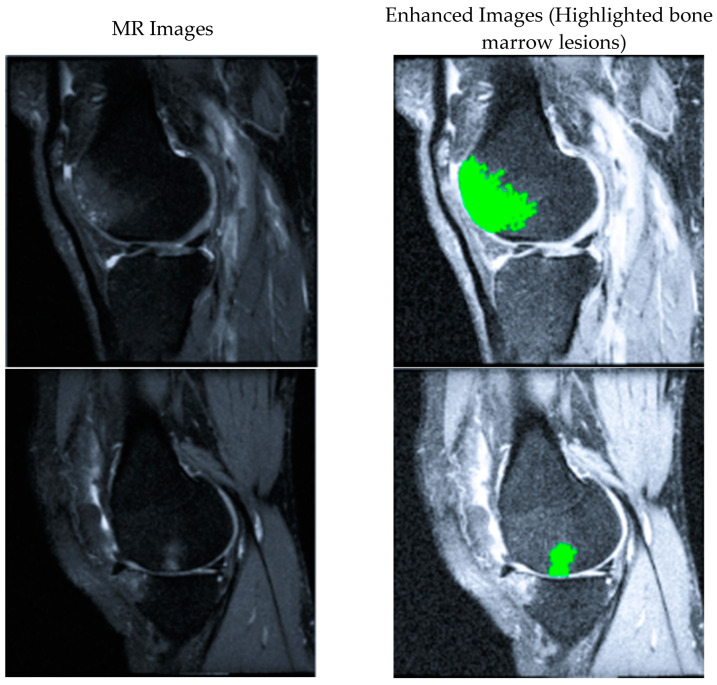
Two examples of MR images with bone marrow lesions (BMLs) marked in green.

BMLs are ill-defined hyperintense regions on fluid-sensitive non-contrast-enhanced MRI [6]. As shown in Figure 1, the brighter regions inside the bones are identified as BMLs. Semi-quantitative methods are commonly used to assess BMLs, which usually grade BMLs into 0–3 grades [4,7,8]. Although previous reports have proved the reader’s reliability of these scoring systems, these systems are subjective and vary among different research. Given the large number of images generated from 3D MRI, the manual quantification of BMLs on each slice image can be tedious. For example, approximately 5 min per scan would require 250 h of reading time for a study on both knees with 500 subjects followed over 3-time points (500 × 2 × 3 × 5 min) [9]. It would be of great benefit to develop an accurate and automatic segmentation method for BMLs. However, BML volume changes in both directions (increase or decrease) at subsequent time points even though volume enlargement is more common than shrinkage [10]. For this reason, the volume change may not be an appropriate metric to validate the performance of the BMLs volume measurement method. 

Over the past decades, MRI has become the prevalent method to evaluate many diseases. Unlike X-rays, the MR signal is sensitive to small changes in the target object, and different types of tissue and biochemical processes, even minimal, can be visualized by adjusting the acquisition parameters. Therefore, MRI could provide detailed and meaningful insight into OA pathology by evaluating the volume of BMLs.

Several studies show BMLs are related to the damage of cartilage or subchondral bone [11,12,13,14,15], and moderate evidence indicates that BMLs are correlated with pain [16]. Before researching the relationship between BMLs and knee OA, one needs to quantify the total volume of BMLs in a knee, which can be achieved by segmenting BMLs in each slice of a knee MRI sequence.

In previous studies, most of the developed BML segmentation methods are semi-automatic. In 2010, Dijkstra et al. used a KNN-based method to segment articular BMLs [17]. They achieved a dice similarity index (DSI) of 0.702 (±0.202) on T2w SPIR MRIs. The method required users to define regions of interest before running the KNN. Each image voxel represents a separate sample, and in the learning set, all voxels were labeled with 0 (non-lesion class) or 1 (lesion class) based on the manual segmentation results. Then, the segmentation result of the BML was obtained in the specified regions of interest. Other studies segmented the BMLs by thresholding the images on signal intensity (SI). Pang et al. proposed a semi-automatic segmentation method in 2013 [18]. They manually defined the regions of interest and computed a threshold of SI through a predefined parameter called false discovery rate (FDR). Spatial features were also included to improve the accuracy of segmentation. The method was not evaluated by comparing it with ground truth but by running intra- and inter-tester reliability tests. The intra-tester reliability was good to excellent for reader 1 (JP; ICC [3,1 model] = 0.79 to 0.99) and reader 2 (GD; ICC [3,1 model = 0.95 to 0.96]); the inter-tester reliability for BML change was good for the lateral femur and tibia as well as the medial femur with the result of ICC [2,1 model] = 0.83 to 0.93, but low for the medial tibia with the result of ICC [2,1 model] = 0.59. Besides using parameters to determine the threshold, Nielsen et al. calculated the average SIs and standard deviations of the manually marked BML regions to define the threshold [19]. Based on their results, the BML volume obtained by the computer-assisted method was significantly correlated with the BML volume measured from the manual delineation. However, the absolute difference between the volumes generated by these two methods was relatively large (1319 mm^3^ vs. 1828 mm^3^ in the femur and 941 mm^3^ vs. 2097 mm^3^ in the tibia). Although the experiment results showed that computer-generated BML volumes were more sensitive to detecting BML change, it still left the accurate and automatic BML segmentation as an open question when manual segmentation is considered as a ground truth.

While semi-automatic bone marrow lesion (BML) segmentation methods are time-consuming, the following study aims to enhance automation. Nonetheless, there remains a scarcity of studies that achieve the fully automatic segmentation of BMLs. In 2013, Dodin et al. proposed a fully automated method [20]. They used an automated bone segmentation method to define the regions of interest, and then segmented BMLs by a threshold and refined the results by mathematical morphology operations (opening and erosion). They achieved a relatively low DSI of 0.49 for the DESS sequence and 0.52 for the IW-TSE sequence in the whole knee. The best DSI achieved was 0.60 for the DESS sequence at the medial anterior of the femur compartment. Stout et al. applied a single reader to semi-automatically segment BMLs [21]. This program requires an operator to mark several points along the boundary of the knee bone to define the area of interest then the boundary of the bone will be segmented automatically. The BML regions will be segmented twice by applying thresholding and curve evolution processes. Two criteria are defined to exclude false positive BMLs: first, the distance between a BML and the articular border should be less than 10 mm; second, a BML should exist on more than one slice. It is proved reliable with the reliability of the intra-tester ICC [3,1 model] baseline BML = 0.86 to 0.97. In 2022, Frank Preiswerk et al. [9] developed and validated a deep learning-based method to segment BMLs in knee MRI data sets using the U-Net model with a dataset of N = 11,676 BML images and mask patch pairs (5673 in training set, 2875 in validation set, and 3128 in test set) and achieved an average 2D Dice Similarity Score of 0.70 and Pearson’s correlation coefficient of 0.94. In this study, they experimented with different resolution levels of U-Net. The model with five resolution levels achieved the best result with a batch size of 64 and using the Dice loss function. This method’s limitation was not as automatic as in the first step; a reader still needs to identify the location of the BML in each image slice with a single mouse click.

The most accurate method to assess BML size may be a detailed image or volumetric segmentation [22,23] but to date, accurate results require substantial user interaction and thus cost an extensive amount of time to complete. The development of fully automatic BML segmentation and volumetric measurement methods is needed to address these limitations.

In this study, we proposed a fully automatic BML segmentation method requiring no human interaction using deep learning models. We compared the proposed method with several other state-of-the-art methods in the same dataset. The rest of the paper is organized as follows: In Section 2, we described our dataset and discussed the proposed method, including the segmentation model and implementation. In Section 3, we presented the experiment setup and analyzed the experiment results along with the evaluation metrics used. Section 4 discussed the overall aim of this study and its limitations, while Section 5 concluded this paper.

## 2. Methods

### 2.1. Dataset

The Osteoarthritis Initiative (OAI) is a multi-center longitudinal study of approximately 4800 adults with or at risk for symptomatic knee OA, sponsored by the National Institutes of Health [24]. The OAI aims to promote the discovery of biomarkers for the development and progression of OA and has developed into one of the state-of-the-art longitudinal databases of images. The OAI owns an institutional review board approval (IRB) from the coordinating centers and four clinical centers (the University of Maryland and John’s Hopkins comprise a single recruitment center, Brown University, Ohio State University, and University of Pittsburgh). All the participants provided informed consent to participate in the OAI. Approximately 4800 people (ages 45–79) with or at risk for knee OA were recruited by the four OAI clinical centers [25]. Both MRI and radiographs are included in the OAI public dataset.

For this study, we took 300 subjects’ MRI scans of the knee from the public dataset. For each subject, two sequences of MRI scans were recorded. One is a sagittal intermediate weighted, turbo spine echo, fat-suppressed (IWFS), and another is a dual echo steady state (DESS). Since the IWFS sequence is more sensitive for identifying subchondral BMLs in size and appearance than DESS, we chose the IWFS sequences in this study. All the images were resized to 448 × 448 for standard input size and normalized with an average of 0 and a standard deviation of 1.

The dataset was randomly split into training, validation, and test sets at 70%, 15%, and 15% at the subject level. Since not every subject has BMLs, and for those subjects with BMLs, not every slice has BMLs, the images without BMLs were excluded. The number of subjects and images in each subset is shown in Table 1.

### 2.2. BML Segmentation Flowchart

**Figure 2:**
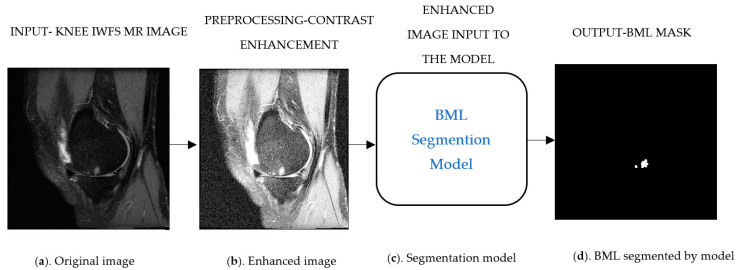
Schematic illustration of bone marrow lesion (BML) segmentation pipeline. (**a**) Original image, (**b**) Enhanced image, (**c**) Segmentation model, and (**d**) BML segmented by model.

As illustrated in Figure 2, the BML segmentation pipeline will take the input as (Figure 2a) MRI IWFS sequence images and the preprocess to enhance its contrast (Figure 2b), this step helps the model to differentiate the underlying structure of the BML with other tissue and noises, and finally the model will generate a corresponding predicted mask (Figure 2d) for each input image. We added up the area of BML from individual slices in each sequence to obtain the BML volume for a subject, then compared the predicted BML volume to the ground truth BML volume to assess the segmentation method.

### 2.3. The Proposed Segmentation Model (U-Net + InceptionResNet-v2 Network)

The proposed segmentation model for BMLs is based on the famous U-Net structure and the InceptionResNet design. Our idea is inspired by the evolving process of classical CNN networks that the network’s performance increases as the model goes deeper. However, going deeper and deeper, the model has the issue of a “vanishing gradient” problem. ResNet was proposed to solve the problem by introducing the residue module [26]. Later work further combined the Inception network and ResNet to generate the InceptionResNet [27]. In this paper, we aim to design a deeper U-Net by introducing more layers and incorporating residual connections to avoid the “vanishing gradient” problem.

The U-Net model is a deep-learning structure proposed by Ronneberger et al. [28]. It is a symmetrical U-shaped network with an encoder, a decoder, and bottleneck paths. The encoder uses fully convolutional blocks to extract relevant features, and these extracted features propagate to the decoder using skip connections. The decoder reconstructs the mask images into desirable dimensions using these feature maps and transposed up convolution. The bottleneck path connects the encoder and decoder paths and contains two 3 × 3 convolution layers. There are four convolution blocks in the encoder path, and each one includes two 3 × 3 convolution layers. The decoder path also has four blocks, each having one 2 × 2 transposed convolution layer, a concatenation layer to connect the extracted feature maps, and two 3 × 3 convolution layers. The final layer is a 1 × 1 convolution layer, mapping every feature vector to the desired number of classes. The output is a binary image as the object mask.

**Figure 3:**
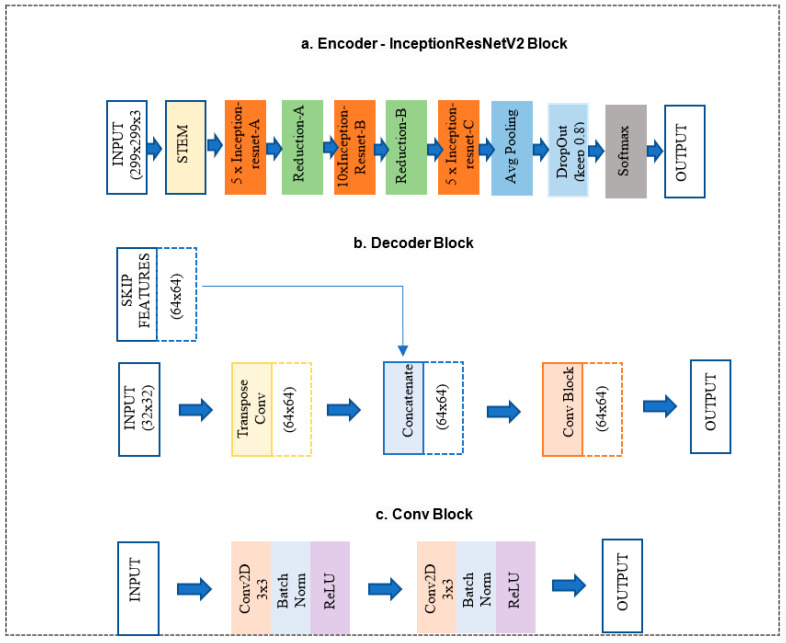
Schema of (**a**) InceptionResNet-v2 encoder block [29], (**b**) decoder block, and (**c**) Conv block.

In the proposed network, the encoder and bottleneck layers of the U-Net architecture were replaced with the InceptionResNet-v2 architecture, as shown in Figure 3a, and decoder paths were replaced with decoder blocks shown in Figure 3b. More details of the two parts are discussed below.

- InceptionResNet-v2 block (Figure 3a) is a convolutional neural network trained on more than a million images from the ImageNet database. The network is 164 layers deep and can classify images into 1000 object categories. Therefore, the network has learned rich feature representations for a wide range of images. The network accepts an image input size of 299 × 299, and the output is a list of estimated class probabilities, as shown in Figure 3a. This block replaced the proposed model’s original U-Net encoder and bottleneck path. Here, we used the pre-trained weights from ImageNet.
- The decoder block (Figure 3b) consists of a 2 × 2 transpose convolution layer followed by the skip connection taken from the InceptionResNet-v2 block encoder, followed by a convolution block (Figure 3c). The convolution block consists of two 3 × 3 convolution layers. A batch normalization layer and a ReLU activation function follow each convolution layer. This block replaced the original U-Net model decoder convolution layer. The Decoder block will take input from the previous block and skip the connection feature map from the encoder to regenerate the segmentation mask using the convolution block.

**Figure 4:**
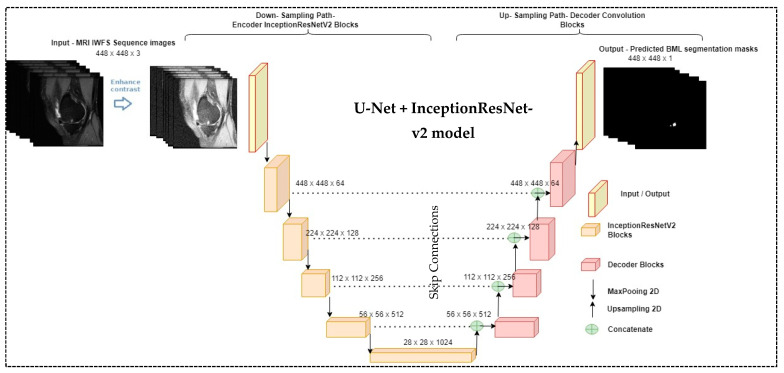
The proposed bone marrow lesion (BML) segmentation method.

Figure 4 illustrates the proposed BML segmentation model, a modified version of U-Net that integrates InceptionResNet-v2. We modified the input filter as 64 × 64 instead of 32 × 32 and changed the final layer output activation function of InceptionResNet-v2 to the Sigmoid instead of the Softmax layer as we predicted the binary mask. The raw MR knee images were pre-processed to enhance the contrast to capture the underlying structure of the BMLs before feeding into the segmentation model. The model output was compared with the manually delineated ground truth for evaluation.

### 2.4. Loss Function

Loss functions play an important role in determining the model performance, and different tasks employ different loss functions. It is decided based on the dataset considering its properties like distribution, skewness, boundaries, etc. BML segmentation aims to predict whether a pixel belongs to the BML mask. Therefore, this problem can be considered as a pixel-wise binary classification problem. Hence, we want to add a binary cross-entropy (BCE) loss function to the commonly used Dice loss function for the BML segmentation model; the BCE loss formula is listed below.

(1) $$BCELoss=-(ylog\left(\hat{y}\right)+(1-y)log\;(1-\hat{y}))$$

where $\hat{y}$ is the prediction of the model and y is the ground truth of the input image. When the observation belongs to pixel 1 the first part of the formula becomes active, the second part vanishes, and vice versa in the case of the observation’s actual pixel being 0.

The Dice score coefficient measures overlap in image segmentation. The advantage of using dice loss is that it can handle the class imbalance in terms of pixel count for the foreground and background. Other generalized loss functions like MSE or BCE do not handle class imbalances. But BCE is much more resilient to both the noise level and the number of pixels changed. It ranges from 0 to 1, with 1 indicating a 100% overlap between the segmented region and the ground truth region, while 0 indicates the two regions have no overlap. The Dice score coefficient formula is given below.

(2) $$2D\;Dice=\frac{2|X\cap Y|}{\left|X\right|+|Y|}$$

The loss function of classic U-Net uses a dice loss function derived from the dice coefficient, and the formula is given below.

(3) DiceLoss=−2D Dice

The dice loss function alone cannot help to improve the model accuracy as the intensity of BMLs is similar to the outer region of bones on MRI. Hence, the model will falsely segment BMLs out of the bone region. It is susceptible to noise, especially when dealing with small tissues like BML and its intrinsic characteristics. As a result, we want to combine generalized loss functions like BCE and specialized loss functions like Dice loss. This allows for some diversity in the loss while benefitting from the stability of BCE and gives us the best result in terms of segmentation, pixel-wise accuracy, generalization, and adversarial attacks. The final loss function used in the proposed segmentation model is given below.

(4) Loss=DiceLoss+BCELoss

## 3. Experiment and Results

### 3.1. Experiment Setup

The distribution of datasets can be found in Table 1. We implemented all the models using Keras [29] with the Tensorflow [30] backend. Hyperparameter optimization was performed for learning rate values ranging from $10^{-2}$ to $10^{-3}$ using the validation set. Based on the models’ performance, the learning rate was set to $10^{-3}$ and the model was trained over 100 epochs with a batch size of 64 and optimized using the adaptive moment estimation (Adam) [31] in NVIDIA RTX 2070 SUPER (Manufacturer: NIVIDIA, 2788 San Tomas Expressway, Santa Clara, CA 95051, USA). EarlyStopping has been used to save training time and avoid model overfitting. It was responsible for stopping the training process if there was no improvement in the accuracy after a specified number of epochs, i.e., 60~75. Since the pre-trained InceptionResnetv2 takes 448 × 448 × 3 as the input dimension and MR images are greyscale images, we converted the greyscale images (one channel) to color images (three channels) using OpenCV 4.5.0 [32] as part of the preprocessing steps before feeding the images to the model.

### 3.2. Evaluation Metrics

We used three metrics to evaluate the model performance and validate the results. They are the 2D Dice Similarity Score (DSC), 3D Dice Similarity Score, and Pearson correlation coefficient.

The 2D DSC is the commonly used measurement for segmentation models. It ranges from 0 to 1, with 1 indicating 100% overlap between the segmented region and the ground truth region, while 0 indicates the two regions have no overlap at all. The 2D DSC formula is shown in Equation (2). Since the 2D Dice score evaluates area overlap at the slice level, it does not provide an overview of the volumetric overlap at the subject level. Therefore, we proposed the 3D DSC to evaluate the volumetric overlap for each subject, as shown in Equation (5). For the numerator part, we counted the common pixels between the predicated BML and ground truth in all slices with BMLs for one subject and added them up. If there is no BML in any of the slices, we do not count that slice. For the denominator part, we added the numbers of the BML pixels in both the ground truth and the predicated masks.

We called this newly proposed evaluation metric 3D Dice Similarity Score (3D DSC) to distinguish it from 2D DSC in the way that 2D DSC evaluates area-based segmentation accuracy at the image level. In contrast, the 3D DSC evaluates volume-based segmentation accuracy at the subject level. The formula of the 3D DICE is listed below:

(5) $$3D\;Dice=\frac{2\sum_{i=1}^{n}\left|Xi\cap Yi\right|}{\sum_{i=1}^{n}\left|Xi\right|+\sum_{i=1}^{n}\left|Yi\right|}$$

where Xi is the predicated BML mask of slice i of a subject and Yi is the corresponding ground truth of slice i, with the slice number starting from i = 1 to n where n is the total number of slices with BML for that subject, and |X| means the total number of non-zero pixels in image X.

The Pearson correlation coefficient (r) [33] is a commonly used evaluation metric in the medicine domain for comparing two methods of measuring the same quantity. It measures the linear relationship between two variables. The correlation coefficients vary from −1 to +1, with positive values indicating the same tendency of the two variables, while negative values indicate the opposite tendency. Correlation coefficient values close to zero indicate a low association between two variables, and those close to −1 or +1 indicate a strong linear relationship between two variables. The correlation coefficient is calculated using Equation (6) below:

(6) $$r=\frac{n\left(\sum xy\right)-(\sum x)(\sum y)}{\sqrt{\left[n\sum x^{2}-{\left(\sum x\right)}^{2}\right]\;\left[n\sum y^{2}-{\left(\sum y\right)}^{2}\right]}}$$

where n is the number of subjects in the testing data set, x represents the BML volume produced by the Deep Learning method and y represents the ground truth of BML volume.

### 3.3. Results and Comparison with Other Methods

We compared our method with another state-of-the-art method developed for BML segmentation in [9] which was based on U-Net. We re-implemented the method in [9] following the same parameter setting specified in the paper and tested their method on our dataset. We also compared the proposed segmentation model with several other segmentation models including U-Net++, Sharp U-Net, and U-Net + ResNet. All the methods in this section were trained and tested on the same training and testing sets specified in Section 2.1. The reported results are from the testing set for all models, as shown in Table 2.

**Figure 5:**
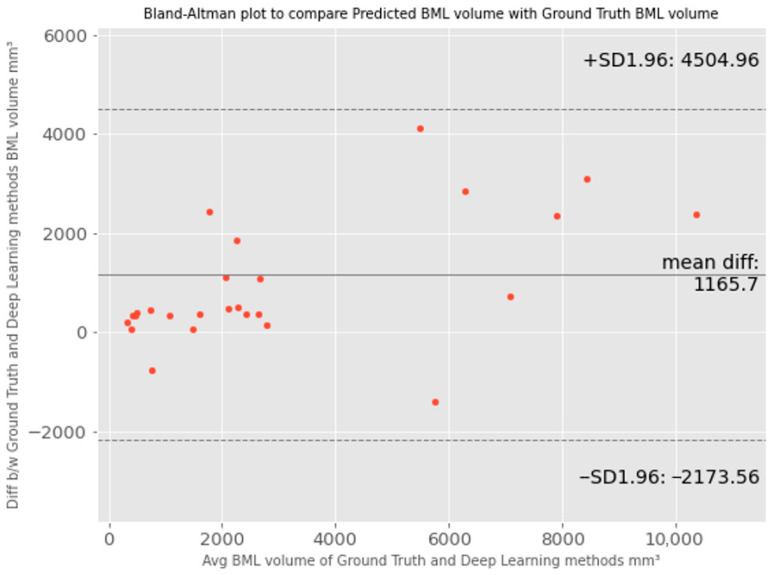
Visualized agreements between predicted bone marrow lesion (BML) volume by the proposed method and ground truth BML volume using the Bland–Altman plot.

We further evaluated the proposed method using Bland–Altman plots, as shown in Figure 5. The Bland–Altman plot compares the difference between two measurements of the same variable [37]. It plots the difference between two measurements against the mean for each pair. The agreement limit (the 95% confidence interval) is defined as 1.96, the standard deviation of the differences between the two measurements. The Bland–Altman plot recommends that 95% of the measurements should lie within the limits of agreement. Therefore, the plot can highlight any anomalies that are points outside the agreed limits. The ground truth BML volume measurement will be the reference method for the proposed BML segmentation method.

From Figure 5, we can see that points are scattered around the mean difference line and within the limit of agreement, and the mean difference (bias) is about 1166 pixels between the ground truth and predicted BML volume. This indicates that the data distribution is generally normally distributed but slightly skewed towards the ground truth method, especially when the bone marrow lesion (BML) volume size is high, i.e., the ground truth BML volumes are larger than the predicted BML volumes, and the discrepancy becomes more pronounced. The larger the average BML volume, the greater the difference between the ground truth and predicted volumes. This discrepancy may be attributed to the fact that Deep Learning models primarily focus on adjacent pixel variation and local features, which may not be readily discernible to humans. Therefore, Deep Learning models tend to draw tighter boundaries than manual delineation by taking into consideration the local features. In this case, ground truth values are overestimated and introduce systematic error or bias. This is one of the issues of the manual BML segmentation process due to the intrinsic characteristics of BML and also an issue during manual segmentation.

**Figure 6:**
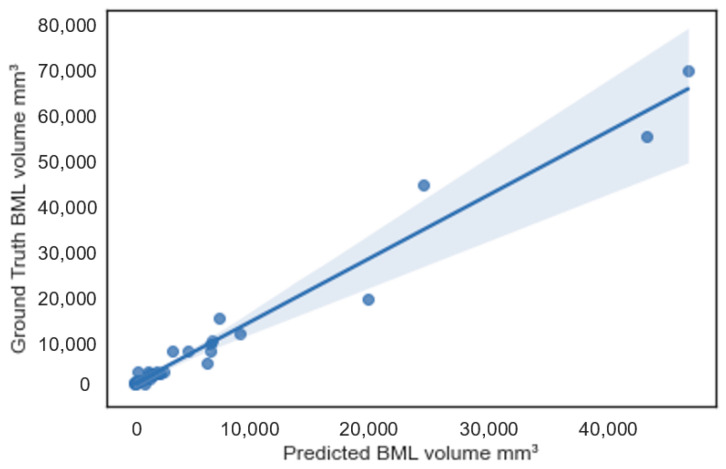
Scatterplot between ground truth and predicted bone marrow lesion (BML) volumes.

Further, in Figure 6, we plotted the PredVol (computer-generated BML volume) and GroundTruthVol (ground truth volume) variables using a regression line plot with a 95% confidence interval. As we can see, the two variables are aligned well with the regression line. Most of the sample points are within or close to the boundary of the 95% confidence interval. This confirms that computer-generated BML volumes are strongly correlated with ground truth BML volumes. Besides, we can also observe that the difference (error) between ground truth and computer-generated BML volumes becomes larger as the BML size increases.

## 4. Discussion

In this study, we proposed an automated segmentation method to measure the volume of BMLs in MRI to facilitate the diagnosis of knee OA. This method has several advantages and innovations compared to existing methods.

- The proposed method is fully automatic with no requirement for human intervention.
- It produces exact BML volume as a quantitative output of the pipeline, which is different from previous methods that generated approximate measures or categorical output, such as manual linear measurement (e.g., the greatest diameter [38], approximate BML volume [12]) or semi-quantitative scoring methods (e.g., BLOKS [7], WORMS [6]).
- It does not require treating different BML sizes differently (e.g., treating larger BML differently for the flow to work [20]). Our method can automatically learn the contextual information about the underlined structures by training different label data variants when the input images pass through the model network. This helps us to achieve full automation and precision for BML segmentation, to facilitate the future incorporation of BML features into the diagnosis model for knee OA.
- Lastly, our method can provide both the 2D slice-level segmentation masks and the 3D volume of BML. This enables evaluation from different levels and perspectives to better understand the model’s performance. The 2D masks may be further used to analyze the shape of BML besides computing the volume only.

We have compared our method with several state-of-the-art methods. These methods include the original U-Net model [9] and a few modified versions of U-Net including U-Net++ [34], Sharp U-Net [35], and U-Net + ResNet34 [36]. As shown in Table 2, the modified versions of U-Net models outperformed the baseline U-Net model on our dataset; however, the best performance was achieved by the proposed method. At the same time, we observed that the accuracy, even with the best model, remains relatively low. Notably, this performance was achieved without any human intervention. One reason for the lower DSC could be attributed to the small size of our dataset, coupled with the diffuse nature of bone marrow lesions (BMLs), which can vary in size, contrast, and shape. The deep learning models need enough variations in training and validation datasets to capture different BML structures. Another reason for the low DSC scores is that BMLs do not have clear and distinctive margins so possible reader bias of the ground truth could be introduced (i.e., the manually labeled BML are consistently larger than the computer-measured BML).

Despite the low DSC accuracy, the high Pearson correlation coefficient r = 0.98 (*p* < 0.05) shows a strong correlation with the ground truth volume. Further, our results show that combining the U-Net model with other classification models as an encoder can generate significantly better performance than the original U-Net, which could be extended to more network architectures and applied to other problems.

While the proposed BML segmentation method has many advantages, there are some limitations and opportunities for improvement. Our dataset was only labeled for BMLs in the femur bones. This should be extended to the tibia and patella bones. Besides, we had a limited number of training and testing samples and used only data from the V00 baseline time point. Further validation and training using different OAI time points and a larger dataset are likely to improve the results. In addition, our method is limited to a segmentation method only, as all the images in our dataset contain BML. The model is not trained to process healthy images without BML. We will include healthy images in the dataset and train the model to process both BML images and healthy images. For healthy images, the corresponding ground truth should be pure black masks.

## 5. Conclusions

A fully automated method to segment BMLs and quantify BML volumes using a proposed Deep Learning model called U-Net + InceptionResNetV2 was proposed in this paper. We achieved a Pearson’s correlation coefficient of 0.98, 2D DSC of 0.68, and 3D DSC of 0.60, using manual BML delineation as ground truth. The method does not require any human intervention and generates both a 2D segmentation mask and a 3D volume measurement. Compared with other state-of-the-art methods, the proposed one outperformed others on the same dataset. This work provides a possible convenient tool to assess BML volumes efficiently in larger MRI data sets to facilitate the assessment of knee OA progression. In future studies, we plan to enlarge the dataset and include BMLs in the tibia and patella bones in addition to femur bones.

## Figures and Tables

**Table 1:** Separation of the dataset into training, validation, and testing sets.

Set	Number of Images	Subjects
Training	1034	210
Validation	194	45
Testing	209	45
Total	1437	300

**Table 2:** Bone marrow lesion (BML) segmentation results from different Deep Learning methods on the same testing dataset.

Model	r (*p* < 0.05)	2D DSC	3D DSC
U-Net-based BML segmentation method [9]	0.96	0.56	0.51
U-Net++ [34]	0.96	0.59	0.58
Sharp U-Net [35]	0.96	0.57	0.53
U-Net + ResNet34 [36]	0.98	0.66	0.59
U-Net + InceptionResNetV2 (proposed)	0.98	0.68	0.60

## Data Availability

No new data were created. Data were pulled from public database OAI (https://nda.nih.gov/oai, accessed on 23 June 2020).
